# *Akkermansia muciniphila* Alleviates Persistent Inflammation, Immunosuppression, and Catabolism Syndrome in Mice

**DOI:** 10.3390/metabo13020194

**Published:** 2023-01-28

**Authors:** Yali Xu, Jianfeng Duan, Dacheng Wang, Jiali Liu, Xiancheng Chen, Xian-Yang Qin, Wenkui Yu

**Affiliations:** 1 Department of Intensive Care Unit, The Affiliated Drum Tower Hospital, Medical School of Nanjing University, Nanjing 210008, China; 2 Laboratory for Cellular Function Conversion Technology, RIKEN Center for Integrative Medical Sciences, Yokohama 2300045, Japan

**Keywords:** chronic critical illness, inflammation, immunosuppression, catabolism, *Akkermansia muciniphila*, gut, microbiome

## Abstract

Many patients in intensive care units, especially the elderly, suffer from chronic critical illness and exhibit a new pathophysiological phenotype: persistent inflammation, immunosuppression, and catabolism syndrome (PICS). Most patients with PICS have a constellation of digestive-system symptoms and gut failure. *Akkermansia muciniphila* (Akk) is a commensal gut bacterium that reduces inflammation, balances immune responses, modulates energy metabolism, and supports gut health. This study investigated the protective effects and underlying mechanisms of live and pasteurized Akk in treating PICS in a mouse model. PICS was induced on day 14 after performing cecal ligation and puncture (CLP) on day 1 and administrating lipopolysaccharide on day 11. Pasteurized or live Akk, or phosphate-buffered saline was administered twice daily by oral gavage for 7 days. Both live and pasteurized Akk attenuated PICS, as evidenced by reduced weight loss, and a reduction in symptoms and serum cytokine/chemokine levels. Liver and intestinal injuries were mitigated, and intestinal barrier integrity improved with Akk administration. Analysis of 16S rRNA amplicon sequences showed that Akk induced significant intestinal microbiota alterations, including increased abundance of Akk, *Muribaculaceae*, *Parabacterbides goldsteinii*, and decreased abundance of *Escherichia_Shigella* and *Enterobacteriaceae*. Collectively, Akk alleviates PICS by enhancing gut barrier function and reshaped the microbial community.

## 1. Introduction

With advances in life support systems, an increasing number of patients can survive lethal multiple organ failure (MOF) in the initial episode of a critical illness; however, long-term survival has not improved significantly [1]. Many survivors, especially patients who are elderly, suffer from severe and prolonged functional disabilities and experience persistent low-grade inflammation and dysregulated immune responses. The devastating pathophysiology is known as “persistent inflammation, immunosuppression, and catabolism syndrome” (PICS) [2]. PICS contributes to poor long-term prognosis in critically ill patients and imposes large financial burdens on families and the healthcare system. Effective treatment protocols are urgently needed. Although the clinical presentation of PICS is prominent, it is difficult to investigate the mechanisms involved and validate relevant treatments in the clinical setting. To elucidate the mechanisms of PICS and explore treatment options, a mouse model was established by conducting a modified sublethal CLP on day 1 and administering low doses of lipopolysaccharide (LPS) on day 11, resulting in critical PICS features on day 14 [3]. The CLP + LPS model involves a composite strike to mimic the main characteristics of PICS in humans. CLP causes severe abdominal infection, and endotoxemia simulated by LPS could further aggravate immune suppression and catabolism. Mice underwent CLP + LPS experienced debilitating damage and repeated inflammation that required rescue treatments, consistent with the physiopathology and clinical characteristics of PICS.

PICS is characterized by a dramatic shift in the composition of bacterial communities in the gut, leading to complications and elevated long-term mortality rates [4]. In addition, the loss of commensal gut microbiota and overgrowth of opportunistic pathogenic flora have been implicated in PICS mice [5]. Over the past decades, there has been increasing interest in modifying the gut microbiome by fecal microbiota transplantation (FMT), probiotics, or prebiotics to improve outcomes in critically ill patients. Probiotics are by far the most widely studied microbiome-based therapeutics to prevent sepsis, the early survivors of which can progress to PICS. Probiotics have been found helpful in reducing ventilator-associated pneumonia, antibiotic-associated diarrhea, and infections in critically ill patients requiring mechanical ventilation [6]. Probiotic intervention in previous studies mostly focused on *Lactobacillus* and *Bifidobacterium* species [7,8]; however, several other next-generation probiotics appear promising in animal studies and preclinical research.

*Akkermansia muciniphila* (Akk), a mucin-degrading bacterium belonging to the phylum Verrucomicrobia, is an intestinal symbiont that colonizes the intestinal mucosal layer of humans and rodents [9]. It is located at the interface between the host and gastrointestinal microbiota, indicating its specific metabolic properties. This symbiont plays a key role in maintaining intestinal health by inducing intestinal adaptive immune responses [10] and promoting epithelial development [11]. Akk is also associated with disease states. Several interventional studies on Akk have revealed its value in improving host metabolic functions [12,13,14] and immune responses [15]. Previous studies have shown an inverse correlation between the abundance of Akk and inflammatory conditions, such as acute appendicitis [16] and inflammatory bowel disease [17,18]. In addition, Akk has been associated with increased sepsis survival in animal models [19], probably because it affects T cell differentiation [10]. These findings indicate that Akk exerts pleiotropic effects on inflammation, modulating energy metabolism and the immune system, which may indicate its capacity to alleviate PICS. Moreover, pasteurized Akk achieves similar or even stronger beneficial effects than live Akk, and both show high safety and tolerability in humans [13,14].

16S rRNA sequencing data from 17 PICS patients revealed a decreased abundance of Akk (0.364 ± 1.11%, not published). Although the role of this bacterium in anti-inflammation, immunomodulation, and energy regulation strongly implies its protective effects against PICS, there have been no published reports to date. Thus, we investigated the protective effects of live and pasteurized Akk against PICS and the potential mechanisms of Akk activity in a mouse model.

## 2. Materials and Methods

### 2.1. Materials and Reagents

Fluorescein isothiocyanate (FITC)-labeled dextran 4 kDa (FD-4) and LPS were purchased from Sigma-Aldrich (St. Louis, MO, USA). Luminex Mouse Magnetic Assay kits were obtained from R&D Systems (Minneapolis, MN, USA). The antibodies of muscle RING finger-1 (MuRF-1), claudin-1, mucin 2 (Muc2), F4/80, GAPDH, β-Actin and myeloperoxidase (MPO) were obtained from Servicebio (Wuhan, China).

### 2.2. Animal Experiments

**Figure 1:**
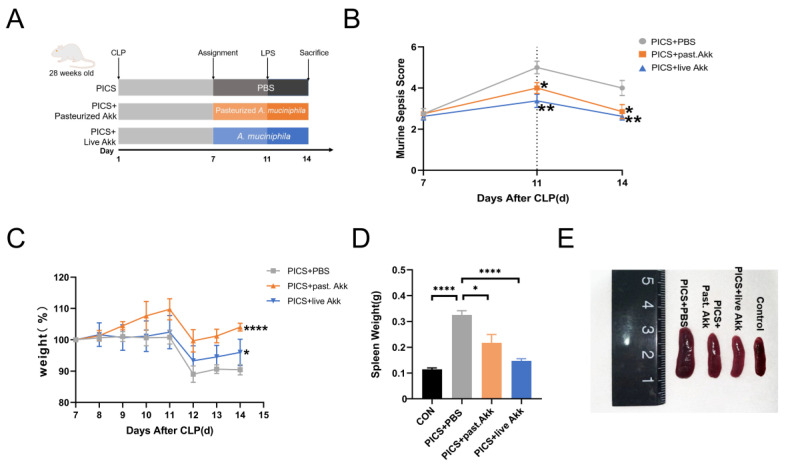
Oral gavage administration of Akk alleviates phenotype in CLP + LPS-induced PICS. (**A**) Establishment of the PICS mouse model and schematic diagram of pasteurized Akk and live Akk treatments. (**B**) MSS calculated with time. (**C**) Body weight changes are expressed as the mean change from the weight on day 7. (**D**,**E**) Representative spleens of four groups (**E**) and the spleen weight (**D**) were analyzed at day 14. CON = control. past. = pasteurized. MSS = murine sepsis score. Data are shown as the mean ± SEM. * *p* < 0.05, ** *p* < 0.01, **** *p* < 0.0001.

Animal experiments were carried out in accordance with protocols approved by the Animal Care and Use Committee of Nanjing Drum Tower Hospital (2022AE01001). Male C57BL/6 mice (28-week-old) were housed in the Animal Research Center of Nanjing Drum Tower Hospital under constant temperature (22 ± 2 °C) and humidity (55 ± 5%) with free access to food and water. A 12-hr day/night cycle was maintained before the formal experiment. Murine models of PICS were established by CLP + LPS as previously described [3]. Briefly, mice in the PICS group were subjected to CLP surgeries on day 1 and injected intraperitoneally with LPS diluted in PBS at a dose of 1 mg/kg on day 11. The PICS states were generated on day 14. Mice in the control group (*n* = 5) underwent the same CLP protocol without ligation and puncture and were intraperitoneally administrated equal amounts of PBS on day 11. As shown in Figure 1A, 24 mice in the PICS group were randomly divided into three groups (PICS + PBS, PICS + pasteurized Akk, PICS + live Akk) on day 7 (*n* = 8 each group). Mice in the PICS + pasteurized Akk and PICS + live Akk groups were gavage-fed pasteurized Akk (1 × 10^8^ colony-forming units (CFU)) or live Akk (1 × 10^8^ CFU) in 0.2 mL PBS solution, respectively, twice a day for 7 days (day 7 to 14). Mice in the PICS + PBS group were administered the same volume of anaerobic sterile PBS via oral gavage. The survival rates, septic severity and body weights were recorded daily throughout the experiment for all mice. Fecal samples were collected on day 7 (before administration of live Akk or pasteurized Akk) and day 14 (the end of the experiments) then stored at −80 °C. On day 14, serum, ileum tissues with fecal materials, liver tissues, spleen tissues, and muscle tissues were collected and stored at −80 °C after the mice were humanely euthanized. A portion of liver and ileum tissues was fixed in Carnoy’s solution for further procedures.

### 2.3. Bacterial Strains and Culture Conditions

Akk strain (ATCC BAA-835) was grown in brain-heart-infusion (BHI) media supplemented with 0.4% porcine mucin (Sigma-Aldrich, St. Louis, MO, USA) and 0.05% cysteine (Sigma-Aldrich, St. Louis, MO, USA) under strict anaerobic conditions (99%N_2_ at 37 °C). The concentration of bacteria was estimated by measuring the absorbance at the wavelength of 600 nm as previously described [20]. In one group of the experiment, Akk was inactivated by pasteurization at 70 °C for 30 min.

### 2.4. Assessment of PICS Severity

According to the Murine Sepsis Score (MSS) system [21], severity of the inflammatory disease for all PICS mice were evaluated on day 7, 11, and 14 after CLP.

### 2.5. Pathological Assessment

Ileum and liver specimens were fixed, embedded in paraffin and cut into sections for hematoxylin and eosin (H&E) staining. After that, an experienced pathologist assessed and scored the pathological changes in the ileum and liver tissue at random locations. A 0–4 grading scale for the histological assessment of ileum injuries was used as previously described [22]. The evaluation of liver injuries used a 0–5 grading scale according to our previous study [3].

### 2.6. Alcian Blue-Periodic Acid-Schif (AB-PAS) Staining

To assess the mucus production and goblet cells, paraffin-embedded ileum sections with fecal materials were prepared and stained with AB-PAS following the manufacturer’s protocol. The tissue slides were then imaged at 20× magnification under a bright field microscope. AB-PAS stains goblet cells within the intestinal villi structure deep purple. The number of goblet cells per intestinal villi were quantified at 4–5 different locations of view, and *n* = 3–5 per group were used for analysis.

### 2.7. Immunohistochemistry (IHC) Analysis

IHC was performed as follows: briefly, 4-µm paraffin-embedded liver and ileum sections were dewaxed and processed for antigen retrieval in sodium citrate for 20 min followed by blocking for endogenous peroxidase in 3% H_2_O_2_ for 15 min. Subsequently, liver sections were incubated with an anti-mouse F4/80 antibody and an anti-MPO antibody overnight at 4 °C to detect the infiltration into the liver of macrophages and neutrophils, respectively. The ileum sections were incubated with an anti-Muc2 antibody. Afterward, the target proteins were visualized by 3, 3-diaminobenzidine (DBA). The MPO positive cells in the liver were counted under high power field (HPF) as previously described. The intensity of Muc2 and F4/80 staining was analyzed using Image J software (1.8.0, National Institutes of Health, Bethesda, MD, USA).

### 2.8. Western Bloting

The extensor muscle and ileum tissues were dissolved in lysis buffer. Proteins were separated by SDS-PAGE and transferred onto polyvinylidene difluoride (PVDF) membranes after being extracted and quantified. The membranes were then incubated with primary antibodies against MURF-1 and claudin-1 overnight at 4 °C and followed secondary antibody incubation. Protein quantification was performed by optical density methods using Image Lab software (Bio-Rad Laboratories, Hercules, CA, USA) and was normalized against GAPDH or β-Actin.

### 2.9. Real-time Quantitative Polymerase Chain Reaction (qRCR)

Total RNA from the ileum tissues was extracted using TRIzol reagent (Invitrogen, Carlsbad, CA, USA) according to the manufacturer’s procedures. Real-time quantitative PCR was performed using the One Step SYBR PrimeScript plus RT-PCR Kit (TaKaRa Biotechnology Company Limited, Dalian, China) in the 7500 real-time PCR system (Applied Biosystems, Waltham, MA, USA). The expression of Gapdh mRNA served as an internal control, and the results were analyzed using the ΔΔCT method. The qPCR primers were synthesized and purified by GENERAY Biotechnology (Shanghai, China), and the following primers were used: for MuRF-1, 5′-GATGGAAACGCTATGGAGAACC-3′ and 5′-ACGGAAACGACCTCCAGACAT-3′; for krüppel-like factor 4 (klf4), 5′-AGGAACTCTCTCACATGAAGCG-3′ and 5′-GGTCGTTGAACTCCTCGGTC-3′; for resistin-like molecule-beta (Relm-β), 5′-CCATTTCCTGAGCTTTCTGG-3′ and 5′-AGCACATCCAGTGACAACCA-3′; and for trefoil factor 3 (Tff3), 5′-CAGATTACGTTGGCCTGTCTCC-3′ and 5′-ATGCTTGCTACCCTTGGACCAC-3′.

### 2.10. Serum Parameter Analysis

The levels of tumor necrosis factor (TNF)-α, interleukin (IL)-6, interferon (IFN)-γ, chemokine (C-C motif) ligand 2 (CCL2), chemokine (C-X-C motif) ligand 2 (CXCL2), and IL-33 in the serum were measured according to the standard procedure of kits (Luminex Mouse Magnetic Assay kits, R&D Systems) and then determined by MILLIPLEX Analyst (V5.1) software (Millipore, Billerica, MA, USA).

### 2.11. In Vivo Intestinal Permeability Measurement

Intestinal permeability was evaluated using the FITC-dextran method, as described previously [23]. Mice were starved for 6 h and then FD-4 (Sigma-Aldrich, 500 mg/kg body weight) was orally gavaged 3 h before sacrifice. The intensity of FITC in the serum was determined by fluorometry (excitation, 485 nm; emission, 520 nm).

### 2.12. 16S rRNA Analysis

Total genomic DNA was extracted from the fecal aliquots (approximately 0.2 g) by a DNA extraction kit (MN NucleoSpin 96 Soi, Macherey-Nagel, Germany). The NanoDrop-2000C spectrophotometer and gel electrophoresis were used to evaluate the quantity and quality of the DNA. Further, the primers 338F (5’-ACTCCTACGGGAGGCAGCA-3’) and 806R (5’-GGACTACHVGGGTWTCTAAT-3’) were used to amplify the V3-V4 hypervariable regions of the 16S rRNA gene, as described previously [24]. The purified amplicons were paired-end sequenced using Illumina MiSeq (Illumina, San Diego, CA, USA). After merging the raw data using FLASH software, the reads were filtered using Quantitative Insights into the Microbial Ecology (QIIME, v1.8.0; http://qiime.org/ (accessed on 11 April 2022)) pipeline. The sequences were clustered into operational taxonomic units (OTUs) with ≥97% identity. A representative sequence for each OUT was screened and aligned.

The Silva database was used as the reference database. A linear discriminant analysis (LDA) of effect size (LEfSe) analysis (https://www.omicstudio.cn/tool/ (accessed on 20 April 2022) was performed to identify the differentially abundant taxon among the experimental groups. Reconstruction of Unobserved States (PICRUSt V1.0.0) was applied to predict the metabolic functions of the microbiota based on the 16S rRNA gene library composition.

### 2.13. Statistical Analysis

Statistical analyses were performed by using GraphPad Prism 8 (GraphPad Software, San Diego, CA, USA). All data are presented as mean ± standard error of the mean (SEM). One-way ANOVA was applied for comparisons between multiple experimental groups, followed by Tukey’s post hoc test. The distribution normalities were estimated by Kolmogorov–Smirnov test. Data with small sample sizes were analyzed using the Krustal–Wallis test, a non-parametric one-way ANOVA. An unpaired Student’s t test was applied for comparison of the two groups with normal distributions. Values of *p* < 0.05 were considered statistically significant differences.

## 3. Results

### 3.1. Administration of Akkermansia muciniphila Alleviates the Phenotype in CLP + LPS-induced PICS

On day 7, 24 mice were randomly selected and divided into three groups: PICS + PBS, PICS + pasteurized Akk, and PICS + live Akk (*n* = 8 for each group). The mice were injected intraperitoneally with LPS at a dose of 1 mg/kg on day 11. On day 14, two mice died in the PICS + PBS group; one mouse died in the PICS + pasteurized Akk group; and no mouse died in the PICS + live Akk group. The murine sepsis score (MSS) decreased with time after CLP and increased after the LPS injection on day 11. The score was much higher in the PICS + PBS group than in the PICS + pasteurized Akk and PICS + live Akk groups on days 11 and 14 (Figure 1B). The mice were weighed daily and the percentage weight change relative to their body weight on day 7 was assessed from day 7 to 14. After intraperitoneal injection of low doses of LPS on day 11 (serving as the second challenge), the mice showed a more significant body weight improvement in the PICS + pasteurized Akk (*p* < 0.0001) and PICS + live Akk groups (*p* < 0.05) than in the PICS + PBS group (Figure 1C). In the PICS mouse model, splenomegaly occurs as a physiological response to systemic inflammation [25]. Owing to CLP and LPS stimulation, the spleens of mice were more enlarged in the PICS + PBS group than in the control group (*p* < 0.0001). After 7 days of pasteurized and live Akk administration, much smaller spleens were observed in the PICS + pasteurized Akk (*p* < 0.05) and PICS + live Akk groups (*p* < 0.0001) (Figure 1D,E).

### 3.2. Degrees of Inflammation and Consumptive Catabolism Are Milder in Akk-treated Mice

**Figure 2:**
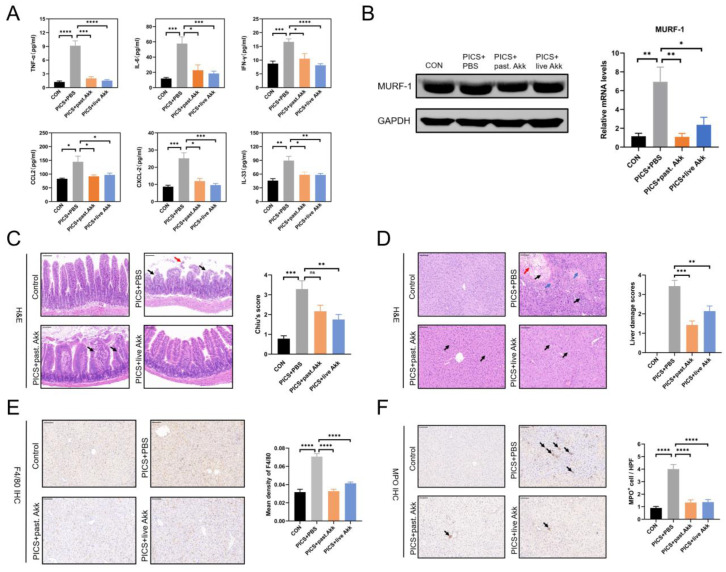
Akk exhibited systemic, hepatic and intestinal anti-inflammatory effects and alleviated hypercatabolism in PICS mice. (**A**) Levels of serum inflammatory indices including TNF-α, IL-6, IFN-γ, CCL2, CXCL2 and a novel biomarker in immunosuppression induction, IL-33, in the four groups. (**B**) The protein (left) and mRNA levels (right) of MuRF-1 were detected by immunoblotting and qRCR. (**C**) Representative H&E staining of ileum tissue. Black arrows indicate separation of intestinal epithelial cells from the lamina propria. The red arrow indicates shedding of intestinal epithelial cells at the villi tip. (**D**) Representative H&E staining of liver tissue. Blue arrows indicate focal necrosis. Black arrows indicate inflammatory cell infiltration. The red arrow indicates hemorrhage. (**E**,**F**) Representative images and quantification of IHC staining of F4/80 (**E**) and MPO (**F**) in liver tissue (indicated by black arrows). Black arrows indicate inflammatory cell infiltration. Scale bars, 100 µm. ns = not significant; Past. = pasteurized. Data are shown as the mean ± SEM (*n* = 5 mice per group). * *p* < 0.05, ** *p* < 0.01, *** *p* < 0.001, **** *p* < 0.0001.

The effects of pasteurized and live Akk on the inflammatory response were investigated in PICS mice at day 14. The blood levels of TNF-α, IL-6, IFN-γ, CCL2, and CXCL2 were measured. TNF-α, IL-6, IFN-γ, CCL2, and CXCL2 levels were increased in the PICS + PBS group compared with those in the control group. Pasteurized and live Akk treatment significantly reduced the release of TNF-α, IL-6, IFN-γ, CCL2, and CXCL2 (Figure 2A). IL-33 is considered an important indicator of sepsis-induced immunosuppression [26]. The levels of IL-33 were higher in the PICS + PBS group than in the control, PICS + pasteurized Akk, and PICS + live Akk groups (Figure 2A). Severe consumptive catabolism with profound muscle wastage has been observed in patients with PICS [27] and in mouse models [3], leading to poor functional outcomes. MuRF-1 contributes to muscle atrophy [28] and showed higher mRNA and protein expression in PICS mice than in the control group, whereas alterations were partially and significantly ameliorated by live and pasteurized Akk treatments (Figure 2B).

The PICS mice exhibited chronic organ damage due to the combined effects of CLP and LPS. Consistent with the beneficial inflammatory profile, H&E staining of the intestine and liver tissues revealed that the damage in mice treated with pasteurized or live Akk was milder, based on tissue morphology and estimation of immune cell infiltration (Figure 2C,D). In detail, CLP and LPS significantly induced the loss of ileum villi as intestinal epithelial cells were separated from the lamina propria (upper right panel of Figure 2C, black arrows) and shed at the villi tip (upper right panel of Figure 2C, red arrow). In contrast, live Akk treatment significantly improved intestinal damage of PICS mice (lower right panel of Figure 2C), while mild separation of the apical mucosal epithelium from the lamina propria was observed (lower left panel of Figure 2C, black arrows), there was no significant detachment observed in the ileum of the PICS + pasteurized Akk group. In the control group, the hepatocytes were regularly arranged with clear hepatic lobules. The liver tissue in the PICS + PBS group displayed cell swelling, focal necrosis (upper right panel of Figure 2D, blue arrows), apparent inflammatory cell infiltration (upper right panel of Figure 2D, black arrows), and hemorrhage (upper right panel of Figure 2D, red arrow). In contrast, only slight liver cell swelling was observed in the PICS + pasteurized Akk and PICS + live Akk groups (lower panels of Figure 2D, black arrows). Macrophages and neutrophils are the major immune cells in MOF and play an indispensable role in promoting the viscous cycle in PICS [28]. Immunohistochemical analysis of the liver tissue revealed that the levels of F4/80^+^ macrophages and MPO^+^ neutrophils were increased in the PICS mice compared with those in the control group. Mice administered Akk (either pasteurized or live) protected macrophage and neutrophil infiltration in the liver of PICS mice (Figure 2E,F). Collectively, these results indicate that Akk attenuated PICS-associated systemic inflammation and immunosuppression, as well as liver injury and small intestinal damage.

### 3.3. Akk Preserves the Intestinal Mucosal Barrier Function in PICS Mice

Next, we explored the mechanism by which Akk prevents PICS. The intestinal mucosal barrier with its chemical and physical barriers plays an important role in maintaining intestinal homeostasis [29]. Intestinal barrier dysfunction contributes to the transition of luminal contents into circulation, thereby inducing inflammation and activating the immune response. Akk supplementation stimulates mucin production [30,31] and improves the integrity of the epithelial cell layer ex vivo [32]. Given the properties described above, we hypothesized that preservation of the intestinal mucosal barrier contributes to the benefits of Akk in PICS.

**Figure 3:**
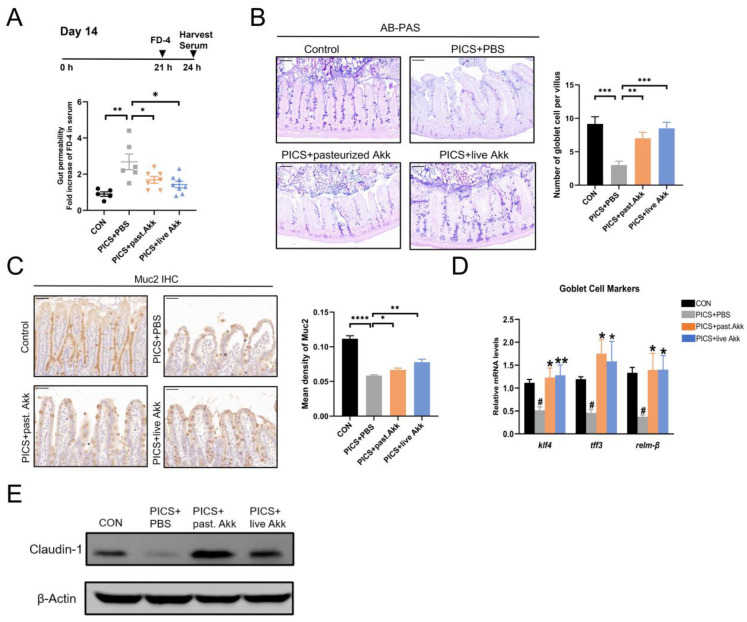
Akk enhanced intestinal mucosal barrier function. (**A**) serum concentration of FD-4 at day 14. (**B**) AB/PAS reaction and the number of acid mucin-producing globlet cells per villus at day 14. Scale bars, 100 µm. (**C**) IHC staining and quantitation of Muc2 in the ileum at day 14. Scale bars, 50 µm. (**D**) Gene expression of goblet cell markers in ileum at day 14. (**E**) Immunoblotting for claudin-1 in ileum at day 14. past. = pasteurized. Data are shown as the mean ± SEM (*n* = 5 mice per group). ^#^ *p* < 0.05, * *p* < 0.05, ** *p* < 0.01, *** *p* < 0.001, **** *p* < 0.0001. * in comparison with the PICS + PBS group, # in comparison with the control group.

Intestinal permeability was evaluated based on the intensity of FD-4 in the blood at day 14. The PICS mice had higher levels of FD-4 in the bloodstream compared with those of the control group, and they were significantly deceased in the mice treated with pasteurized and live Akk (Figure 3A). This data suggests pasteurized and live Akk could reduce intestinal permeability in PICS mice. Subsequently, mucus thickness and goblet cells in the ileum tissue sections were evaluated using AB-PAS staining. The pasteurized and live Akk treatments significantly ameliorated the production of mucus and the reduction in goblet cells compared with the PICS + PBS group (Figure 3B).

Then, the effects of pasteurized and live Akk on goblet cell maturation and function in PICS mice were evaluated. Muc2 secreted by goblet cells is a major component of the ileal mucus barrier. Muc2 expression was examined using IHC. The expression of Muc2 in the ileum was downregulated in the PICS + PBS group compared with that of the control group and moderately but significantly upregulated in the PICS + pasteurized Akk and PICS + live Akk groups (Figure 3C). Compared with the PICS + PBS group, the PICS + pasteurized Akk and PICS + live Akk groups exhibited increased expression of klf4, a zinc-finger transcription factor positively correlated with goblet cell differentiation [33] (Figure 3D). Tff3 and relm-β are important mucosal defense factors secreted by goblet cells [34]. Tff3 cooperates with Muc2 to enhance the protective barrier properties of the intestinal mucosa, whereas Relm-β plays an important role in immunity regulation and host defense against intestinal inflammation. Relm-β and Tff3 expression levels were increased in the PICS + live Akk and PICS + pasteurized Akk groups compared with those in the PICS + PBS group (Figure 3D).

As markers of tight junction structure, claudin-1 was detected in ileum tissue using Western blotting. The protein levels of claudin-1 were lower in the PICS + PBS group than in the control group. However, the levels of the protein increased in the PICS + live Akk group and dramatically increased in the PICS + pasteurized Akk group (Figure 3E).

### 3.4. Akk Reshapes the Gut Microbiota of PICS Mice

Finally, the composition, abundance, and function of gut microbiota in fecal samples were analyzed using high-throughput sequencing of the V3–V4 regions of the 16S rRNA genes. Paired fecal samples were collected from three groups (PICS + PBS, *n* = 4; PICS + pasteurized Akk, *n* = 5; PICS + live Akk, *n* = 7) on day 7 (before intervention; baseline) and day 14 (after intervention; endpoint). A total of 2,557,182 clean reads were obtained for further analysis, and 2412 identified operational taxonomic units (OTUs) were clustered with a 97% similarity cutoff.

**Figure 4:**
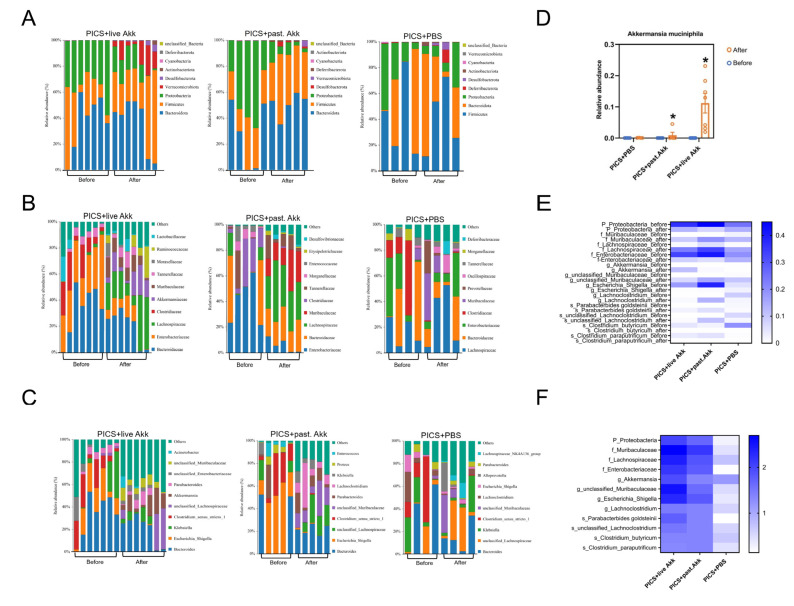
Pasteurized and live Akk reshapes the gut microbiota community. (**A**–**C**) Bar chart of fecal bacterial community composition at the phylum (**A**), family (**B**) and genus (**C**) levels at day 14. (**D**) Relative abundance of Akk at the genus level before and after treatment at day 14. (**E**) Heatmap of the statistically different intestinal microbiota after pasteurized and live Akk treatment and PBS in PICS mice at day 14. (**F**) A heatmap generated by the -log(*p*-value) was drawn to show the alterative levels of the microbial community in Figure 4E. past. = pasteurized. Data are shown as the mean ± SEM. * *p* < 0.05, in comparison with the same group before treatment.

The structure of the intestinal flora can differ significantly even in mice with similar physiological status. In that case, we evaluated the alteration in the intestinal microbiota from baseline to endpoint to minimize the effect of individual differences. To investigate whether Akk administration modulates the structure and composition of intestinal microbiota during PICS, the Wilcoxon rank sum test at three different taxonomic levels was used to compare the distribution of bacteria between day 7 and 14 in the three groups. The histograms illustrate the species and relative abundance of intestinal microbiota at the phylum level (Figure 4A). Consistent with a previous study, *Firmicutes* and *Bacteroidota* were the most abundant phyla in all mouse fecal samples [17]. The abundance of *Proteobacteria* was decreased in the PICS + pasteurized Akk (*p* < 0.05) and PICS + live Akk group (*p* < 0.05). At the family level (Figure 4B), the relative abundance of *Lachnospiraceae* (*p* < 0.01) and *Muribaculaceae* (*p* < 0.01) increased greatly, and *Enterobacteriaceae* (*p* < 0.01) decreased after live Akk treatment. *Lachnospiraceae* (*p* < 0.05) and *Muribaculaceae* (*p* < 0.01) were also increased due to the pasteurized Akk treatment. In addition, *Enterobacteriaceae* (*p* < 0.05) was decreased in the PICS + pasteurized Akk group.

The abundances of intestinal microbiota at the genus level were also analyzed. As shown in Figure 4C, both pasteurized and live Akk administration increased the abundance of Akk (*p* < 0.05), unclassified *Muribaculaceae* (*p* < 0.05 and *p* < 0.01 respectively), and *Lachnoclostridium* (*p* < 0.05) and decreased the abundance of *Escherichia_Shigella* (*p* < 0.05 and *p* < 0.01 respectively). There were no significant changes in the PICS + PBS group. The abundances of the genus Akk in the three groups on day 7 (before gavage) and day 14 (after gavage) suggest that Akk could colonize the colon 7 days after twice daily intragastric administration of 10^8^ Akk cells (Figure 4D). These obvious alternations in intestinal microbiota confirmed the regulatory effect of Akk on the gut microbiota. The changed genera might be important regulatory bacteria in the process of both pasteurized and live Akk exerting their effects.

To identify differentially abundant intestinal microbiota, linear discriminant analysis effect size (LEfSe) analysis was used to explore specific bacterial phylotypes associated with the live and pasteurized Akk treatments (Supplementary Figures S1–S3). Higher abundances of potentially beneficial microbes such as *Parabacterbides goldsteinii* were observed both in the PICS + pasteurized Akk and PICS + live Akk groups. A hierarchically clustered heat map analysis based on LefSe analysis was constructed to identify the intestinal bacterial communities with the same trend of significant changes in PICS + pasteurized Akk and PICS + live Akk groups (Figure 4E). The upregulated potentially beneficial microbes, such as Akk, *Parabacterbides goldsteinii* and *Muribaculaceae*, and down-regulated opportunistic pathogenic bacteria, such as *Gammaproteobacteria*, *Enterobacteriaceae* and *Escherichia_Shigella*, were observed after both live and pasteurized Akk treatment but not after PBS treatment (Figure 4F).

**Figure 5:**
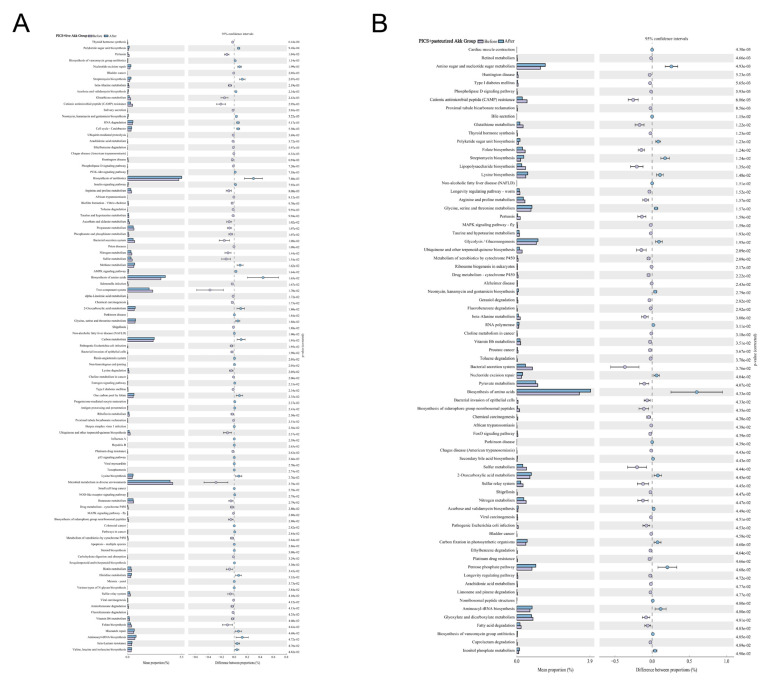
Pasteurized and live Akk regulates metabolic pathways of microbial communities. Fecal microbial community function differences against the KEGG database on Level 3 before and after (**A**) live Akk and (**B**) pasteurized Akk administration at day 14. For *p*-value, the letter e is used to mean “10 to the power of”.

To further explore the biological functions, gut microbiome metabolic functions were predicted at the KEGG pathway Level 3 (Figure 5). The capability for bacterial invasion of epithelial cells, vitamin B6 metabolism, and some inflammatory pathways, for example, the MAPK signaling pathway, were significantly changed after treatment with pasteurized and live Akk. No significant functional differences were observed in the PICS mice treated with PBS.

## 4. Discussion

Recent studies have demonstrated that the decreased or lack of abundance of Akk is highly linked with increasing inflammation in the context of multiple diseases, such as Crohn’s disease, ulcerative colitis, HIV, diabetes and obesity [12,13,14,15,16,17,18]. Moreover, a similar finding suggests that both live and pasteurized Akk could restore LPS-mediated intestinal barrier damage [35]. However, the therapeutic effects of Akk against PICS have not been demonstrated. Here, we investigated the impacts of Akk (live and pasteurized) and the underlying mechanisms in a CLP + LPS-induced mouse model of PICS. Our study showed that both live and pasteurized Akk administration in CLP + LPS-induced PICS mice reduced aggravated inflammation and increased muscle catabolism; reduced hepatic inflammation, liver injury, and intestinal injury; preserved the mechanical and chemical barrier function of the intestinal mucosa; and reshaped the gut microbiota composition in the mice.

In previous studies, higher levels of inflammatory cytokine/chemokine in serum were observed in patients with PICS [36] and in a mouse model of PICS [3]. Increased levels of cytokines (TNF-α, IL-6, and IFN-γ) and chemokines (CCL2 and CXCL2) are the main characteristics of PICS [3]. Our results demonstrated that Akk treatment alleviated the inflammatory response by inhibiting systemic pro-inflammatory cytokines and chemokines in mice with PICS. In addition, IL-33, which has been reported to play a major role in the induction of long-term immunosuppression [26], showed significant relief in the PICS + live Akk and PICS + pasteurized Akk groups, suggesting that Akk may have an important effect on immune dysfunction in mice with PICS. Profound muscle atrophy and weight loss are common in PICS patients during their hospitalization despite the nutritional support [37]. The expression of MuRF-1 is upregulated in critically ill patients and contributes to muscle wasting [38]. In the present study, CLP + LPS markedly upregulated MuRF-1 expression at both transcriptional and translational levels, which was recovered by treatment with Akk.

Patients with PICS are subject to protracted organ dysfunction and recurrent nosocomial infections, and develop a vicious, self-stimulating cycle. The gastrointestinal tract has long been considered to play a crucial role in the pathophysiology of the acute phase of PICS and functions as a driver of multiple organ dysfunction syndrome [39]. Traditionally, intestinal injury and compromise in the intestinal barrier function facilitate the translocation of bacteria and exogenous pathogen-associated molecular patterns (PAMPs), with subsequent induction of proinflammatory pathways and distant-organ dysfunction [39,40]. PICS patients often suffer from different degrees of gastrointestinal dysfunction leading to reduced absorption of nutrients [41]. In addition, the construction and function of intestinal microbiota is demonstrated to be significantly changed in PICS patients and subsequently affects the organism’s metabolism [42]. The state of malnutrition could impair immune cell metabolism and suppress the host’s immune response [43]. In this regard, gut microenvironment dysfunction may act as a significant initial event that results in the vicious cycle of PICS. Akk emerged as sentinel of the gut and its protective effects on inflammation-related conditions by restoring gut permeability have been extensively described. Western diet-induced inflammation and circulating endotoxin levels have been prevented by Akk treatment through the upregulation of intestinal tight junction proteins [44]. Akk can improve dextran sulfate sodium-induced ulcerative colitis and protect gut barrier function [17]. Moreover, Akk can reduce intestinal permeability and alleviate inflammation, thereby stimulating bone fracture healing [20]. Consistent with these findings, our study showed that the intestinal mucosal barrier function was compromised in the PICS mice. Akk restored the intestinal mechanical and chemical barrier, as evidenced by the lower level of FD-4 in the serum, normalized mucus thickness, increased number of goblet cells, and upregulated expression of tight-junction proteins (claudin-1). The results indicate that Akk may exert protective anti-inflammatory effects on PICS through restoring the intestinal microenvironment. Interestingly, we observed similar protective effects of both pasteurized and live Akk against PICS. This is possibly due to the fact that the outer membrane of Akk could retain biological activities after pasteurization [14], suggesting the critical role of the composition of the outer membrane, such as Amuc_1100 [15], on systemic inflammation and hypercatabolism.

To date, the mechanisms that are responsible for the improved gut barrier caused by Akk are controversial. Akk protected gut barrier function by reshaping the microbial community in a colitis mouse model [17]. However, a study on overweight and obese humans found that supplementation with either live or pasteurized Akk did not affect the overall structure of the gut microbiome [13]. Consistent with this finding, alteration of the gut microbiota was not observed in mice supplemented with live Akk [45]. This variation in the shift of the microbiota composition could be the result of sample size and the species involved in the experiments. Evaluation strategies such as pairing individuals from baseline to endpoint and group comparisons at the end of interventions may also contribute to the variation. In the present study, we provided evidence that live and pasteurized Akk treatment increased the abundance of *Muribaculaceae*, *Lachnospiraceae*, Akk, *Lachnoclostridium*, and *Parabacterbides goldsteinii*, whereas it reduced the abundance of *Proteobacteria*, *Enterobacteriaceae*, *Escherichia_Shigella*, *Clostridium_butyricum*, and *Clostridium_paraputrificum*. These effects may explain the benefits of live and pasteurized Akk on PICS mice. *Muribaculaceae* abundance is negatively correlated with inflammation status and could tolerate the immunity stimulation by inhibiting CD8^+^ T cell activation [46]. Members of *Lachnospiraceae* and *Lachnoclostridium* are among the main producers of health-promoting short-chain fatty acids [47,48]. The bacterium *Parabacterbides goldsteinii* was recently reported as a novel probiotic associated with enhanced gut integrity and reduced inflammation [49]. An abnormal expansion of the bacterial phylum *Proteobacteria* is a marker for dysbiosis and a diagnostic criterion for disease [50]. In addition, an expansion of the *Enterobacteriaceae* family is also frequently associated with gut inflammation [50,51]. *Escherichia_Shigella* is an adherent-invasive bacterium and reported to cause gut microbiota disturbance in the development of colitis [52]. The decreased abundance of *Clostridium_butyricum* and *Clostridium_paraputrificum* in the PICS + pasteurized Akk and PICS + live Akk groups seems to conflict with the conclusion. Previous studies have shown that *Clostridium_butyricum* produces butyrate and is safely used as a probiotic [53,54]. These alternations could be the results of interactions between the intestinal microorganisms.

Taken together, this study demonstrated that pasteurized and live Akk administration alleviated PICS-related symptoms, systemic inflammation, hypercatabolism, and immunosuppression; inhibited hepatic and intestinal damage; and restored intestinal mucosal barrier function. The mechanism of enhanced barrier function could be the regulation of the structure of intestinal microbiota through reductions in the abundance of pathological bacteria (*Proteobacteria*, *Enterobacteriaceae*, and *Escherichia_Shigella*) and increases in the abundance of beneficial bacteria (*Muribaculaceae*, *Lachnospiraceae*, Akk, *Lachnoclostridium*, and *Parabacterbides goldsteinii*).

## 5. Conclusions

In summary, this study reveals a protective role for Akk as a critical regulator of chronic systemic inflammation. Live and pasteurized Akk could regulate fecal gut microbiota community and promote intestinal mucosal barrier function to alleviate PICS associated inflammation and multiple organ dysfunction. Thus, Akk as a potential therapeutic agent holds promise for the treatment of systemic inflammation, immunosuppression, and hypercatabolism syndrome.

## Data Availability

The datasets involved in this study are available through the NCBI home page: www.ncbi.nlm.nih.gov/sra/?term=PRJNA916125 (released on 28 February 2023).

## Supplementary Materials

The following supporting information can be downloaded at: https://www.mdpi.com/article/10.3390/metabo13020194/s1. Figure S1: Discriminative biomarkers with an LDA score >4.0 in the PICS + live Akk group before (blue) and after (orange) intervention (left); the LEfSe cladogram represents the taxa enriched before (blue) and after (orange) intervention (right). Figure S2: Discriminative biomarkers with an LDA score >4.0 in the PICS + pasteurized Akk group before (blue) and after (orange) intervention (left); the LEfSe cladogram represents the taxa enriched before (blue) and after (orange) intervention (right). Figure S3: Discriminative biomarkers with an LDA score >4.0 in the PICS + PBS group before (blue) and after (orange) intervention (left); the LEfSe cladogram represents the taxa enriched before (blue) and after (orange) intervention (right).
