# Supplementary material for: Akkermansia muciniphila Alleviates Persistent Inflammation, Immunosuppression, and Catabolism Syndrome in Mice
Source: Metabolites. 2023 Jan 28;13(2):194. doi: 10.3390/metabo13020194 (PMC9961567; doi:10.3390/metabo13020194)
Supplement: Supplementary file 1 [file metabolites-13-00194-s001.zip › metabolites-2155503-supplementary.pdf]

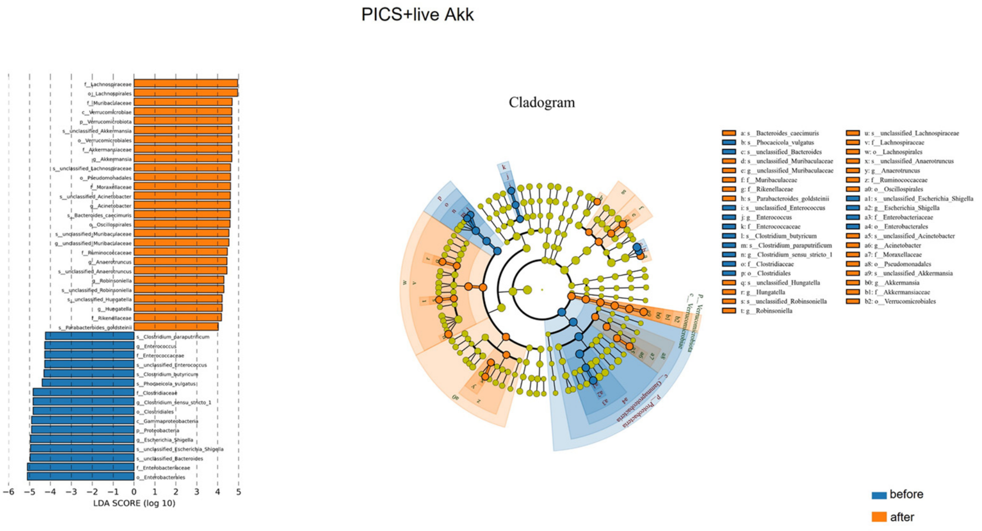

Figure S1. Discriminative biomarkers with an LDA score >4.0 in the PICS + live Akk group before (blue) and after (orange) intervention (left); the LEfSe cladogram represents the taxa enriched before (blue) and after (orange) intervention (right).

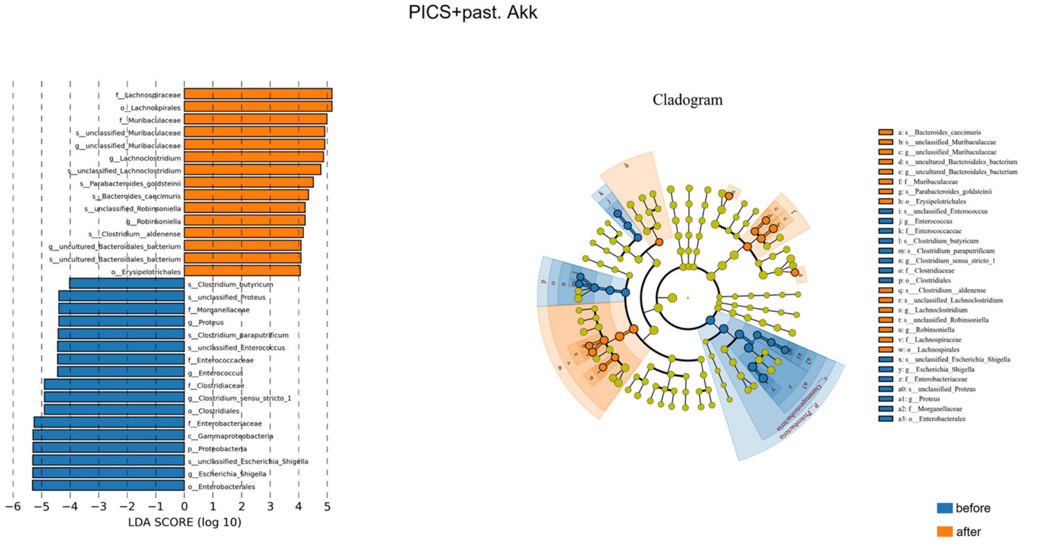

Figure S2. Discriminative biomarkers with an LDA score >4.0 in the PICS + pasteurized Akk group before (blue) and after (orange) intervention (left); the LEfSe cladogram represents the taxa enriched before (blue) and after (orange) intervention (right).

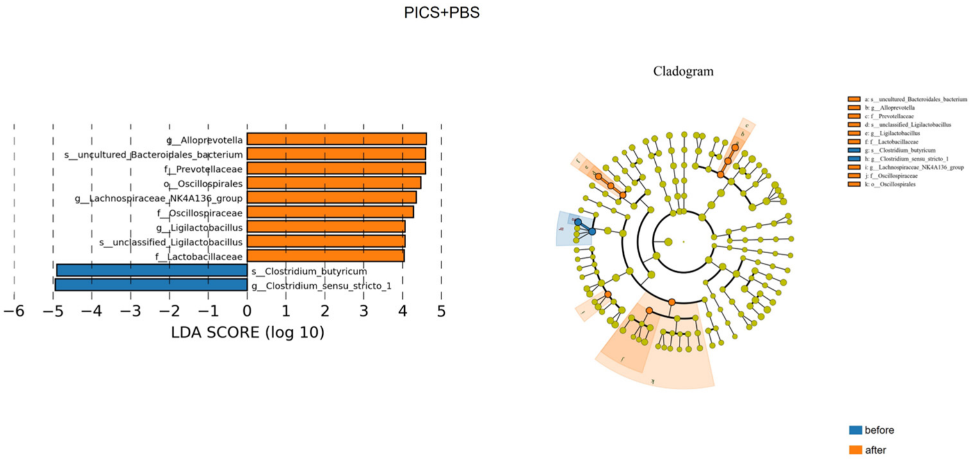

Figure S3. Discriminative biomarkers with an LDA score >4.0 in the PICS + PBS group before (blue) and after (orange) intervention (left); the LEfSe cladogram represents the taxa enriched before (blue) and after (orange) intervention (right).
